# Oxygen-Glucose Deprivation Induced Glial Scar-Like Change in Astrocytes

**DOI:** 10.1371/journal.pone.0037574

**Published:** 2012-05-22

**Authors:** Rongrong Wang, Xiangnan Zhang, Jianxiang Zhang, Yanying Fan, Yao Shen, Weiwei Hu, Zhong Chen

**Affiliations:** 1 Department of Pharmacology, Key Laboratory of Medical Neurobiology of Ministry of Health of China, Zhejiang Province Key Laboratory of Neurobiology, School of Basic Medical Sciences, College of Pharmaceutical Sciences, Zhejiang University, Hangzhou, Zhejiang, People’s Republic of China; 2 Department of Pharmacology, Shanxi Medical University, Taiyuan, Shanxi, People’s Republic of China; 3 Zhejiang Provincial Key Laboratory of Medical Genetics, School of Life Sciences, Wenzhou Medical College, Wenzhou, Zhejiang, People’s Republic of China; Biological Research Centre of the Hungarian Academy of Sciences, Hungary

## Abstract

**Background:**

It has been demonstrated that cerebral ischemia induces astrocyte reactivity, and subsequent glial scar formation inhibits axonal regeneration during the recovery phase. Investigating the mechanism of glial scar formation will facilitate the development of strategies to improve axonal regeneration. However, an *in vitro* model of ischemia-induced glial scar has not yet been systematically established.

**Methodology and Principal Findings:**

In the present study, we at the first time found that oxygen-glucose deprivation (OGD) *in vitro* can induce rat cortical astrocytes to present characteristics of glial scar. After OGD for 6 h, astrocytes showed a remarkable proliferation following 24 h reperfusion, evaluated by 3-(4,5-dimethylthiazol-2-yl)-2,5-diphenyltetrazolium bromide assay and BrdU immunocytochemistry. Meanwhile, the expression of glial fibrillary acidic protein significantly increased, so did the expression of neurocan, which is a hallmark of the glial scar. In further experiments, neurons were co-cultured with astrocytes, which had been exposed to OGD, and then the immunostaining of class III β-tubulin was carried out to assess the neurite growth. When the co-culture was performed at 48 h reperfusion of astrocytes, the neurite growth was obviously inhibited, and this inhibition could be reversed by chondroitinase ABC, which digests glycosaminoglycan chains on CSPGs, including neurocan. However, the processes of neurons were elongated, when the co-culture was performed immediately after OGD.

**Conclusions and Significance:**

Our results indicated that after conditioned OGD the astrocytes presented the characteristics of the glial scar, which are also comparable to the astrocytes in acute and chronic phases after cerebral ischemia *in vivo*. Therefore, the present system may be used as an *in vitro* model to explore the mechanisms underlying glial scar formation and the treatments to improve axonal regeneration after cerebral ischemia.

## Introduction

Cerebral ischemia can lead to astrocyte activation and glial scar formation [1], [2], [3]. Recent studies suggest that glial scar has a dual role in central nervous system (CNS) repair after stroke [4], [5], [6]. In the acute phase following ischemic injury, glial scar formation is crucial for sealing the site of injury and remodeling the tissue, and temporally and spatially controlling the local immune response [6]. On the other hand, the glial scar, a barrier against neurite growth, prevents the recovery of CNS function in the chronic phase [7], [8]. Thus, a detailed understanding of the glial scar, including its formation and changes over time, is crucial to develop effective therapeutic strategies for stroke patients.

Reactive astrocytes are the main cells consisting the glial scar, although microglia are also included [6], [9]. The processes of astrocyte activation in response to CNS injuries have specific structural and functional characteristics. Reactive astrocytes upregulate expression of glial fibrillary acidic protein (GFAP) and various extracellular matrix molecules, and begin proliferating rapidly [5], [10]. The extracellular matrix molecules present the main barrier against axonal growth, which predominantly contain chondroitin sulfate proteoglycans (CSPGs), such as neurocan and phosphacan [8].

So far, little is known about the mechanisms underlying glial scar formation, and few models of the glial scar *in vitro* have been established. It has been reported that cultures stretched by abrupt deformation of the silastic culture plates produce scar-like reactive astrocytes [7]. In another model [11], reactive astrogliosis gradually forms when injured by either a mechanical scrape or foreign-body placement (segments of 50 µm diameter stainless steel microwire). Some factors such as ATP, EGF, and TGF, play an important role in the astrogliosis *in vitro* and *in vivo*, however, whether the glial scar induced by such factors *in vitro* closely mimics that after ischemia remains doubt, since there may exist complex system to induce the glial scar after ischemia [12]–[14]. So, there is no ideal *in vitro* model to mimic the glial scar induced by cerebral ischemia, and to describe the acute and chronic phase changes of astrocyte after cerebral ischemia.

In the present study, we found that oxygen-glucose deprivation (OGD) *in vitro* can induce astrocytes to present characteristics of glial scar, which play a dual role in neurite growth of neurons.

## Results

### Effect of OGD and Reperfusion Time on Reactive Gliosis of Astrocytes

**Figure 1:**
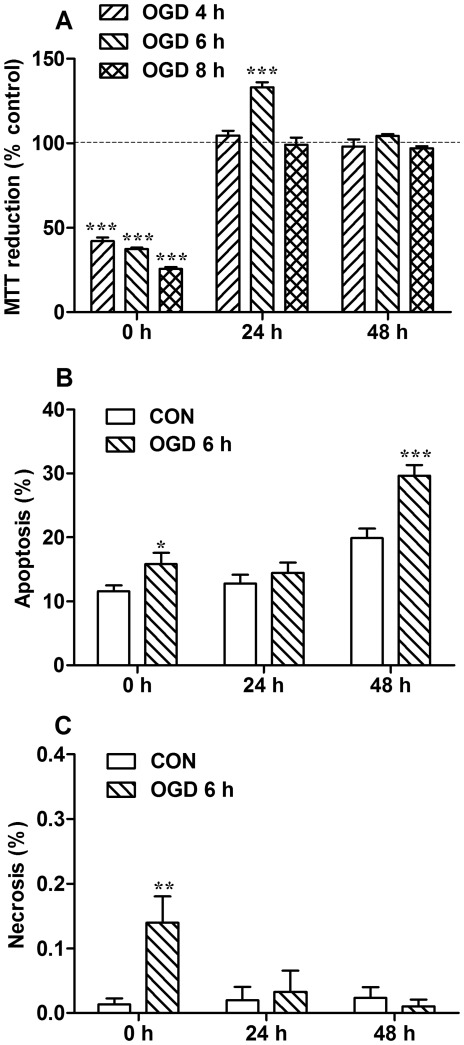
The effects of different OGD durations on MTT reduction and apoptosis and necrosis in astrocytes. Cells were exposed to OGD for 4, 6 or 8 h, followed by reperfusion for 0, 24 or 48 h. The percentage of MTT reduction, apoptosis and necrosis was assayed after various durations of reperfusion. Values are expressed as percentage of control values and are from 3 to 6 independent experiments. * *P*<0.05; ** *P*<0.01; *** *P*<0.001, compared with control group.

**Figure 2:**
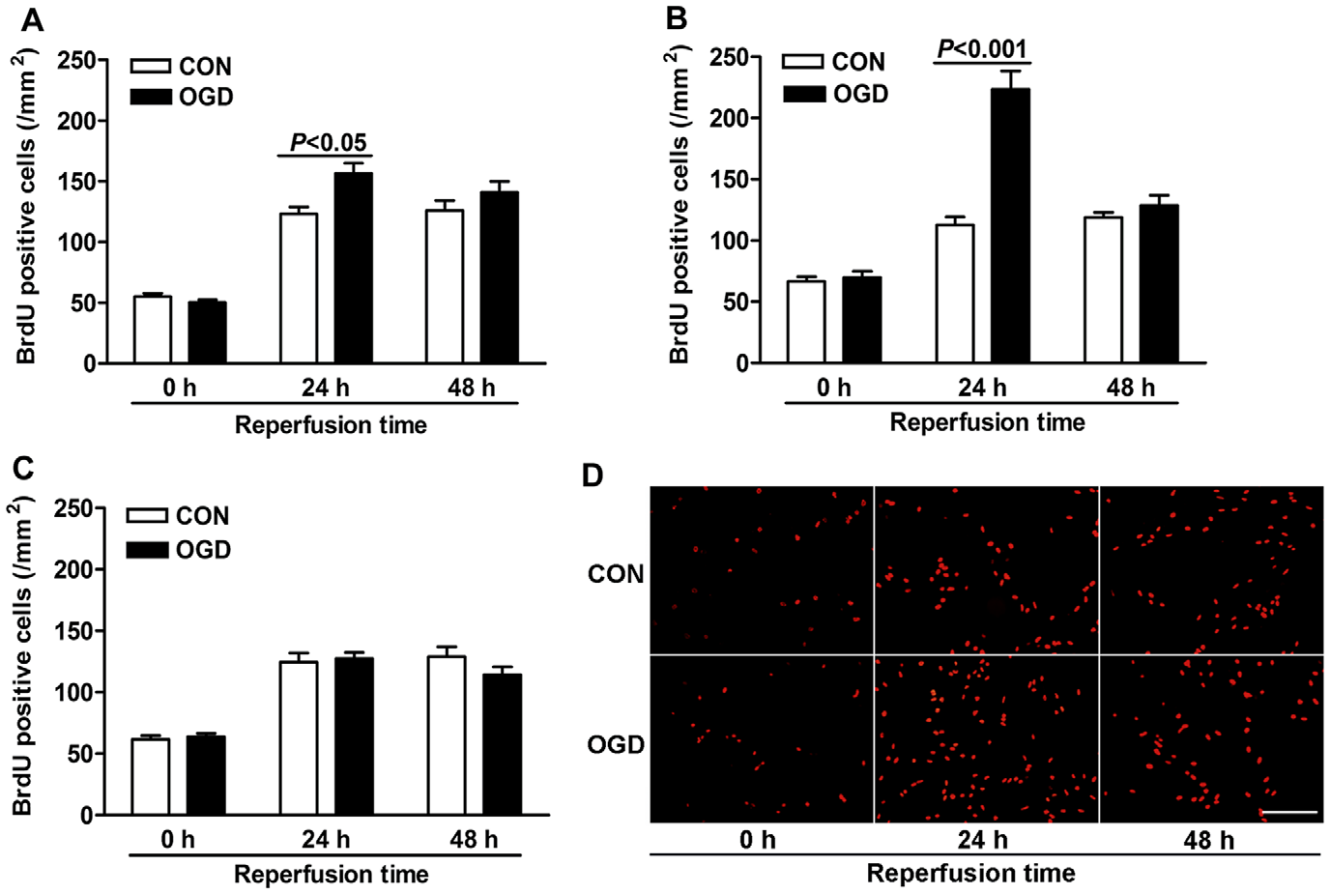
Proliferation of astrocytes were analyzed at various reperfusion times after OGD. Numbers of BrdU positive cells after BrdU immunostaining (red) were qualified after 4 h (A), 6 h (B) or 8 h (C) OGD followed by indicated durations of reperfusion. The BrdU positive cells after 6 h OGD were shown in (D). Scale bars = 200 µm. Values are from 3 to 6 independent experiments.

The effects of different OGD durations on 1-(4,5-dimethylthiazol-2-yl)-3,5-diphenylformazan (MTT) reduction were assessed after different reperfusion times. We found that the MTT reduction of astrocytes returned to control level when exposed to 2 h OGD followed by 24 h reperfusion. When exposed to OGD for 12 h followed by 24 h reperfusion, the MTT reduction was only 20% of controls, which indicated such exposure was too severe (data not shown). Further, we tested three intermediate durations of OGD, which were 4, 6 and 8 h, respectively. At acute phase (0 h reperfusion), OGD for 4, 6 or 8 h caused a significant decline of MTT reduction (Fig. 1A), indicating an obvious decrease of viability at these conditions. However, after 24 h reperfusion, only 6 h of OGD caused a significant increase of MTT reduction, which was possibly due to the proliferation. After 48 h reperfusion, the MTT reductions of all the three OGD-treatment groups were not significantly different from their corresponding controls. We further investigated whether the changes of MTT reduction were contributed by the proliferation or death. Astrocytes only showed obvious increase of proliferation, by BrdU^+^-labeling, at 24 h reperfusion after OGD (Fig. 2). When reperfused for 24 h after 4 h OGD, the number of proliferating cells in OGD group was 123% of that in control group (Fig. 2A). While reperfused for 24 h after 6 h OGD, the number of proliferating cells notably increased to nearly double of the amount of controls (Fig. 2B, D). Thus, the duration of 6 h for OGD was used in the following experiments, as it induced the most obvious astrogliosis. Then cell apoptosis and necrosis condition was analyzed by Hoechst 33342 and propidium iodide double staining after 6 h OGD (Fig. 1B, C). At acute phase (0 h reperfusion) and 48 h reperfusion after 6 h OGD, the percentage of apoptosis significantly increased compared with controls, but not at 24 h reperfusion after OGD. No signs of obvious necrosis were detected at any of the time points after OGD, although it (only about 0.14%) showed a significant increase at acute phase.

**Figure 3:**
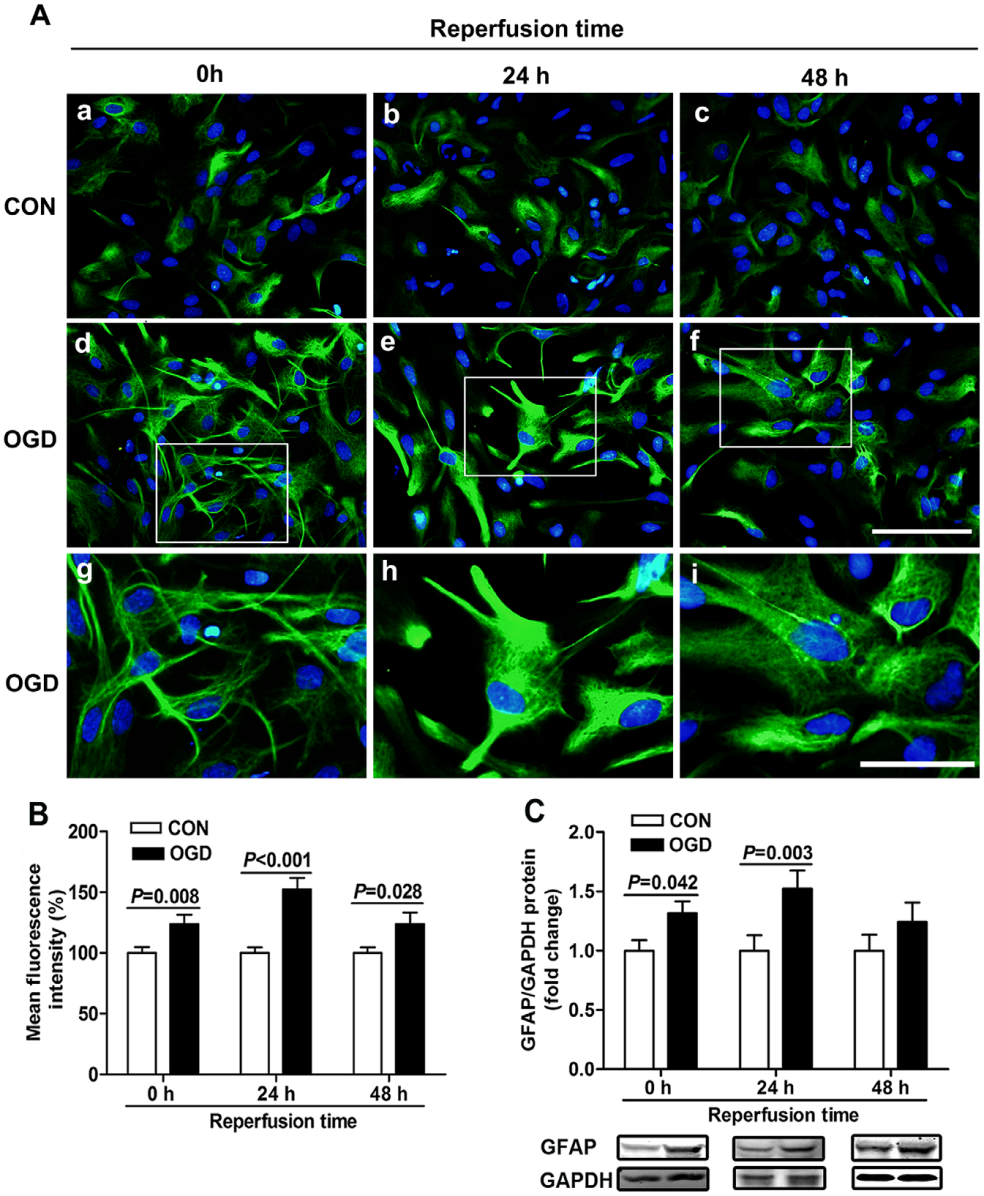
GFAP expression and proliferation of astrocytes were analyzed at various reperfusion times after 6 h OGD. (A) GFAP immunostaining (green) after OGD. Control astrocytes cultured for 0 (a), 24 (b) and 48 h (c) were flat and polygonal. Astrocytes treated with OGD became stellate and clustered, extended processes, and had high GFAP fluorescence intensity in stretched processes after 0 h reperfusion (d, box in d is enlarged in g). After 24 h reperfusion, astrocytes were hypertrophic, and GFAP staining intensity was upregulated in cell body and processes (e, box in e is enlarged in h). And after 48 h reperfusion, some astrocytes still had high GFAP fluorescence intensity in their cell body or processes (f, box in f is enlarged in i). Figures g, h and i were amplification of boxes in figures d, e and f, respectively. Scale bars = 100 µm for a-f; and scale bars = 50 µm for g-i. (B) Quantification of mean GFAP fluorescence intensity. (C) Western blot analysis of GFAP expression. Values are from 3 to 6 independent experiments.

To ensure that OGD caused reactive astrocytes, we did the GFAP immunocytochemistry staining at different durations of reperfusion after OGD. The astrocytes became stellate and clustered, and had extended processes in the acute phase (0 h reperfusion) in response to OGD (Fig. 3A (d, g)), while the astrocytes in controls showed a flat and polygonal morphology (Fig. 3A (a)). The GFAP fluorescence intensity also increased remarkably by 24% (control: 100.0±4.8%, OGD: 123.8±7.4%), especially in stretched processes (Fig. 3A (g), B). At 24 h after OGD, the astrocytes became hypertrophic (Fig. 3A (e, h)), and had high GFAP fluorescence intensity in their cell body and processes. Quantitative analysis showed that the mean fluorescence intensity of GFAP in the OGD groups was 52% higher than that in the control groups (control: 100.0±4.5%, OGD: 152.4±9.1%) after reperfusion for 24 h (Fig. 3B). After reperfusion for 48 h, some astrocytes still had high GFAP fluorescence intensity in their cell body or processes (Fig. 3A (f, i)), and by quantitative analysis the immunoreactivity of GFAP in OGD groups remained 24% higher than control groups (Fig. 3B). The Western blot analysis was also performed to confirm the high expression of GFAP after OGD (Fig. 3C). We found that the expression of GFAP was upregulated after OGD similar as the immunocytochemistry results, but only with significance at 0 h and 24 h after OGD.

### OGD Upregulated Neurocan but Not Phosphacan

**Figure 4:**
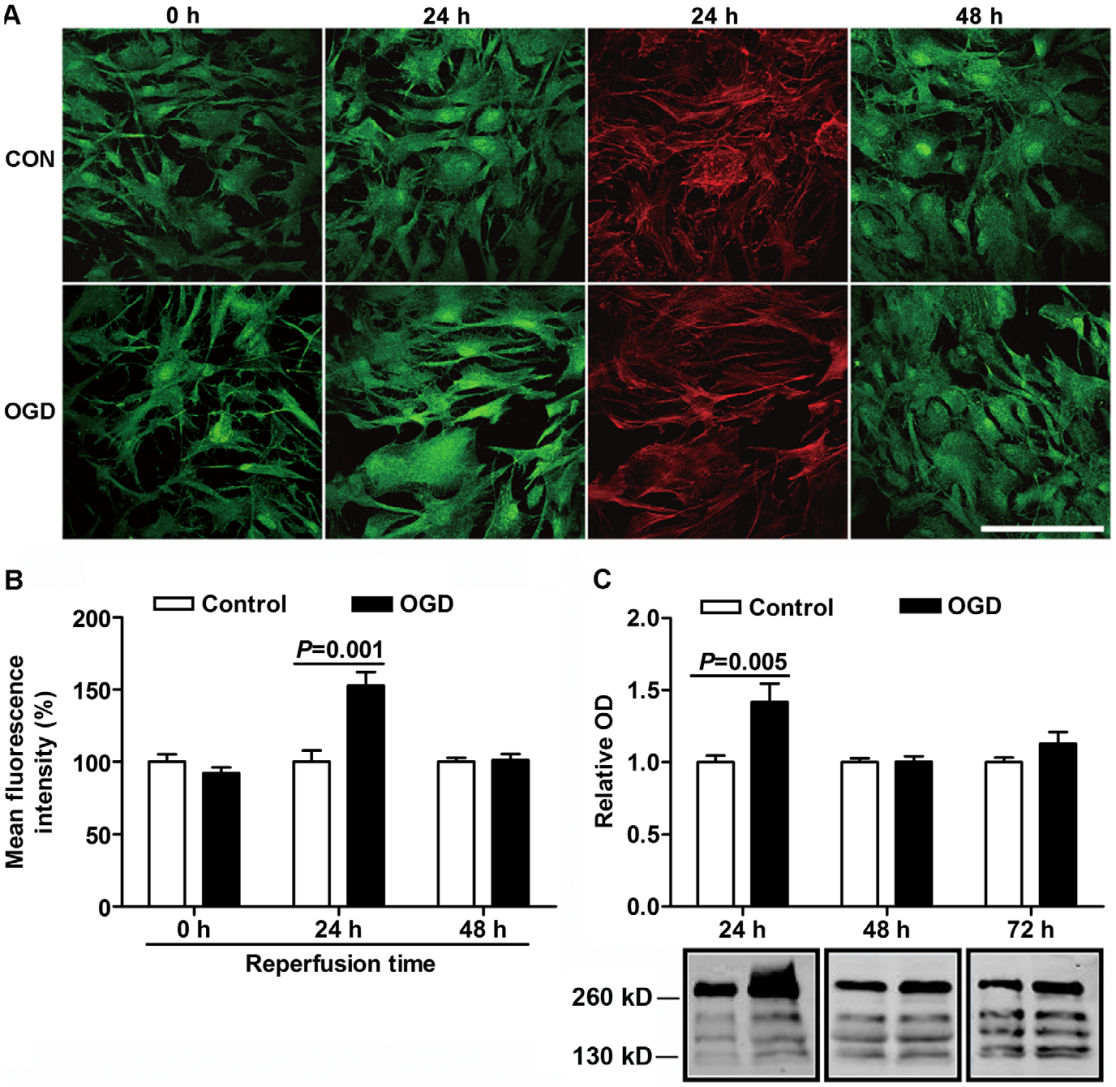
Immunocytochemistry staining and Western blot analysis of neurocan at various reperfusion times after 6 h OGD. (A) Neurocan (green) and actin (red) staining after OGD followed by indicated durations of reperfusion. Scale bars = 100 µm. (B) Quantification of mean neurocan fluorescence intensity. (C) Western blot analysis of neurocan secretion in conditioned medium from astrocytes (major core bands are about 130, 150, 190, and 270 kD). Values are expressed as percentage or fold change of control values and are from 3 to 6 independent experiments.

**Figure 5:**
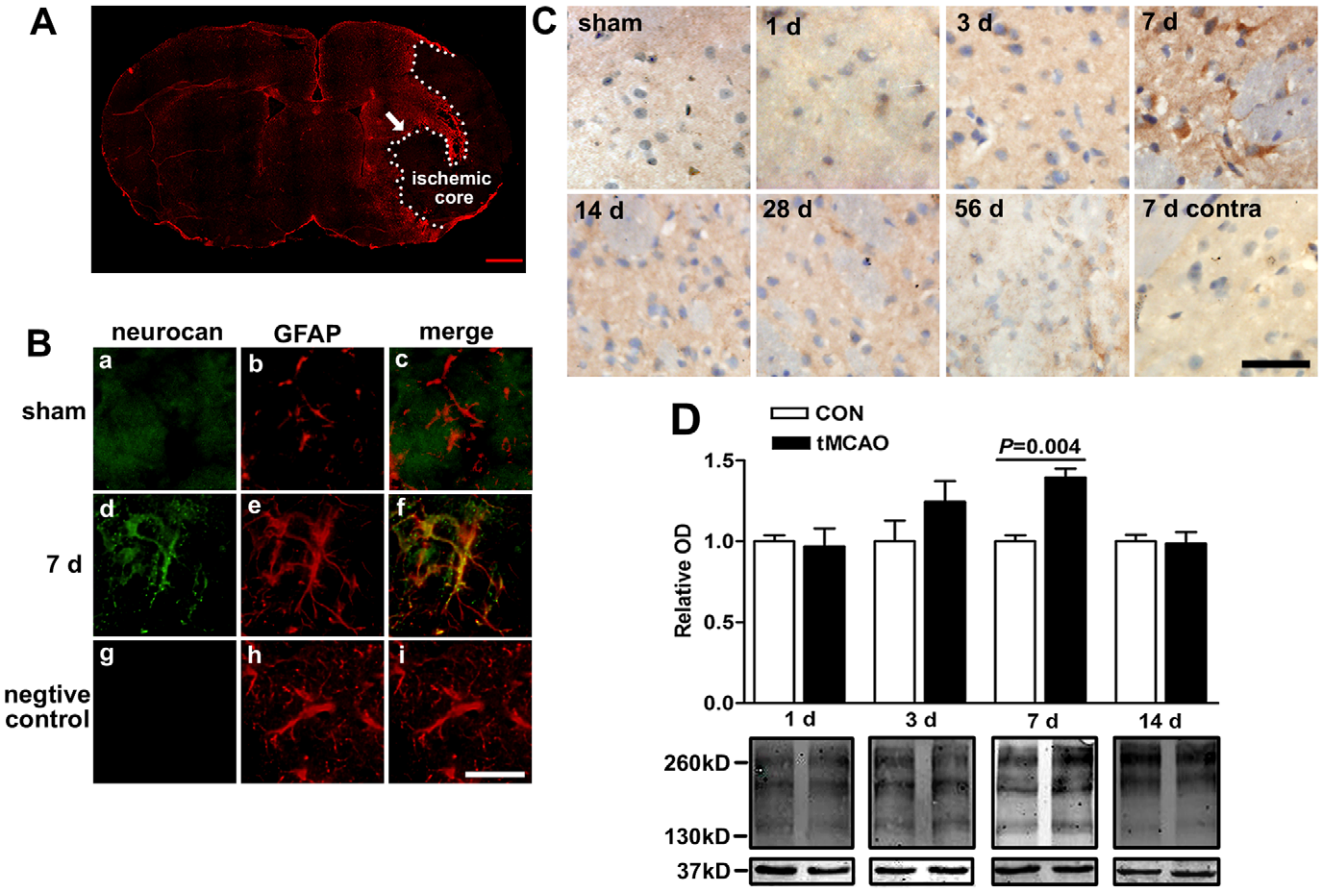
Effect of cerebral ischemia on neurocan expression in the penumbra region in rats. Fluorescent double immunostaining of neurocan and GFAP at 7 d after tMCAO in rats were performed (GFAP: red; neurocan: green; B (d-f) was the enlarged image from the penumbra region indicated by arrow in A; B (a-c) showed the image of the same region in sham group). The negative control by doing the same immunohistochemistry procedure without incubating with anti-neurocan was shown in g-i. DAB histochemistry staining of neurocan at different reperfusion duration after tMCAO were performed and the image from the penumbra region or contralateral (contra) region are shown in C. (D) Western blot analysis of neurocan expression (major core bands are about 150, 190, and 270 kD), which was normalized by GAPDH (∼37 kD). A: scale bars = 1 mm; B, C: scale bars = 25 µm. Values are expressed as percentage or fold change of control values and are from 3 to 6 rats.

To further determine if OGD caused glial scar formation, we evaluated the expression of the main scar-associated proteins, neurocan and phosphacan, at different time points after OGD. The expression of neurocan by immuocytochemistry assay showed no differences between OGD and control groups in the acute phase after OGD (Fig. 4A). However, the mean fluorescence intensity of neurocan was notably upregulated by 53% in OGD groups compared to control groups at reperfusion for 24 h (Fig. 4B). Meanwhile, the expression of actin still remained the same as controls (control: 100±6.1%; OGD: 95.6±12.4%; n = 5, *P*>0.05), suggesting the upregulation of neurocan was not the result of false-positives but because of OGD (Fig. 4A). Secreted neurocan levels from the conditioned medium were also increased at 24 h after OGD (control: 1.00±0.05, OGD: 1.42±0.13) by Western blot analysis (Fig. 4C). After 48 h reperfusion, the expression of neurocan in the OGD groups returned to the control values by immunocytochemistry analysis and Western blot analysis (Fig. 4B, 4C). To confirm the change of neurocan *in vitro* was similar as that *in vivo* after ischemia, the expression of neurocan in the penumbra region was analyzed by immunostaining and Western blot in rats. Seven days after ischemia induced by tMCAO, the expression of neurocan showed obvious elevation with the activation of astrocytes indicated by GFAP staining, but its expression decreased to the level of control at 14 d after ischemia (Fig. 5). The double positive yellow areas were present on astrocytic cell bodies or processes at 7 d after tMCAO, which suggested that astrocytes expressed neurocan after cerebral ischemia (Fig. 5B).

**Figure 6:**
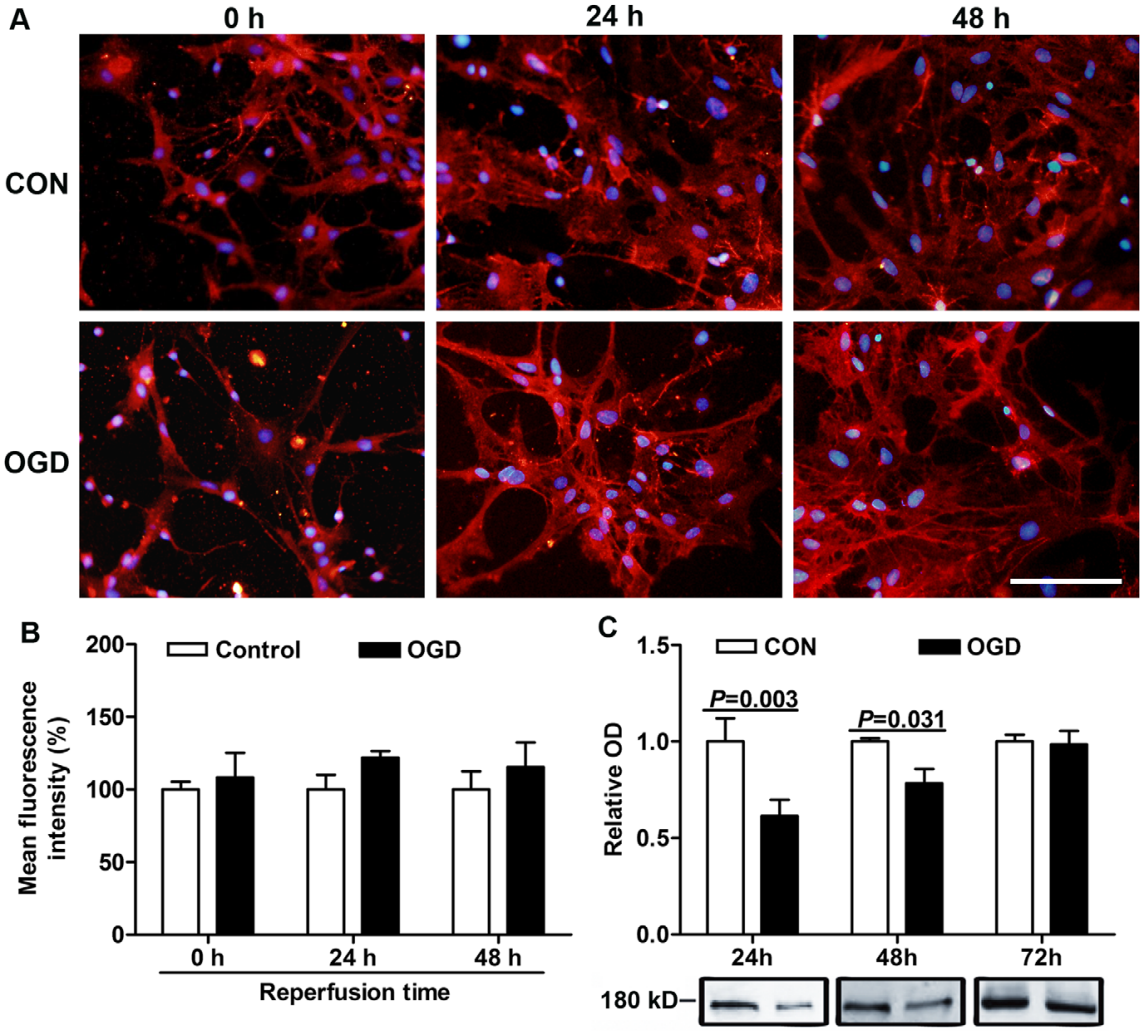
Immunocytochemistry staining and Western blot analysis of phosphacan at various reperfusion times after 6 h OGD. (A) Phosphacan staining after OGD followed by indicated durations of reperfusion. Scale bars = 100 µm. (B) Quantification of mean phosphacan fluorescence intensity. (C) Western blot analysis of phosphacan secretion in conditioned medium from astrocytes. Values are expressed as percentage or fold change of control values and are from 3 to 6 independent experiments.

The expression of phosphacan in astrocytes showed no significant changes between OGD-treated and control astrocytes after OGD by immunocytochemistry assay (Fig. 6A, B). Phosphacan secretion was also assessed in conditioned medium from controls and OGD treated astrocytes. To our surprise, phosphacan secretion was dramatically decreased at 24 h after OGD to only 61% of controls (Fig. 6C). After 48 h reperfusion, it was reduced to 78% of controls, and then gradually recovered to control levels at 72 h reperfusion (Fig. 6C).

### Neurite Growth were Promoted in Acute Phase but Inhibited after 48 h Reperfusion by Reactive Astrocytes

**Figure 7:**
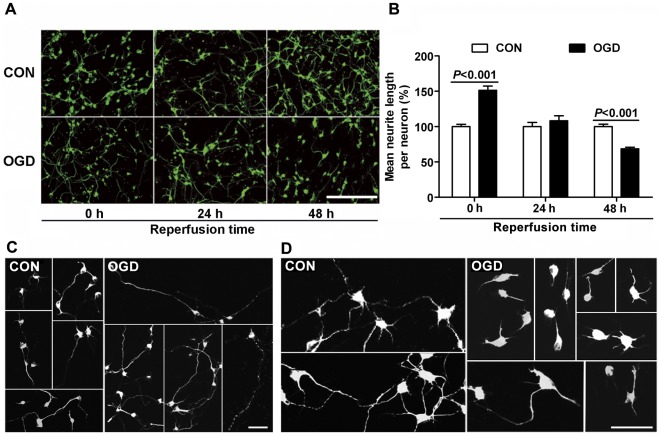
Effect of co-culture with astrocytes on neurite growth in physically separated model (N model). (A) class III β-tubulin immunostaining of embryonic cortical neurons after co-culture with astrocytes for 24 h. Before co-culture, astrocytes were subjected to 6 h OGD or not followed by indicated durations of reperfusion. Scale bar = 200 µm. (B) Quantification of mean neurite length per neuron. (C) Representative figures of class III β-tubulin immunostaining of embryonic cortical neurons co-cultured with astrocytes for 24 h from 0 h reperfusion. Scale bar = 50 µm. (D) Representative figures of class III β-tubulin immunostaining of embryonic cortical neurons co-cultured with astrocytes for 24 h from 48 h reperfusion. Scale bar = 50 µm. Values are from 3 to 6 independent experiments.

**Figure 8:**
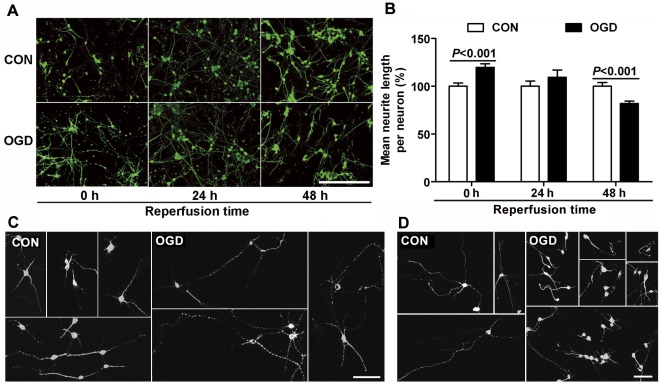
Effect of co-culture with astrocytes on neurite growth in directly contacted model (A+N model). (A) Class III β-tubulin immunostaining in embryonic cortical neurons after co-culture with astrocytes subjected to 6 h OGD or not followed by indicated durations of reperfusion. Scale bar = 200 µm. (B) Quantification of mean neurite length per neuron. (C) Representative figures of class III β-tubulin immunostaining of embryonic cortical neurons co-cultured with astrocytes for 24 h from 0 h reperfusion. Scale bar = 50 µm. (D) Representative figures of class III β-tubulin immunostaining of embryonic cortical neurons co-cultured with astrocytes for 24 h from 48 h reperfusion. Scale bar = 50 µm. Values are from 3 to 6 independent experiments.

**Figure 9:**
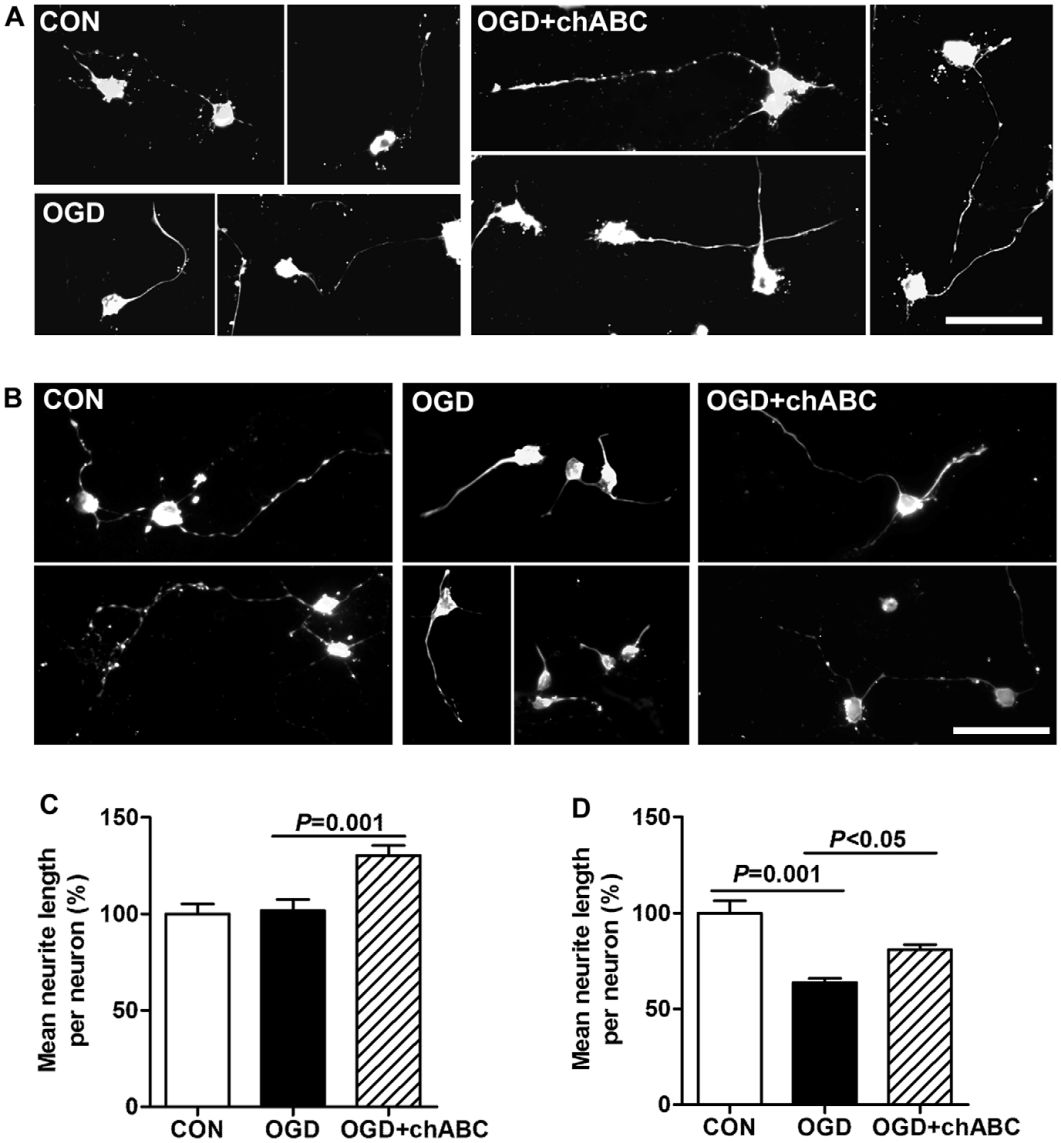
Effect of co-culture with astrocytes on neurite growth after treated with chABC in physically separated model (N model). Representative figures of class III β-tubulin immunostaining of embryonic cortical neurons after co-culture with astrocytes for 24 h from 24 h (A) or 48 h (B) reperfusion. chABC at 0.2 U/ml was added into the astrocytes at 2 h before co-culture. Quantification of mean neurite length for neurons after co-culture with astrocytes for 24 h from 24 h (C) or 48 h (D) reperfusion. Scale bar = 50 µm. Values are from 3 to 4 independent experiments.

To study the effect of reactive astrocytes on neurite growth, cultured neurons were seeded on astrocytes at three different times after OGD. When neurons were co-cultured with astrocytes immediately after OGD, the neurites grew longer than controls, in both the physically separated N model (OGD: 151.4±6.1%, control: 100.0±3.2%, Fig. 7A-C) and the directly contacted A+N model (OGD: 119.7±3.7%, control: 100.0±3.4%, Fig. 8A-C), whereas there were no remarkable changes in the number of neurites between controls and co-cultured groups (data not shown). When co-culture was performed at 24 h after OGD, the promoting effect on neurite growth disappeared. The mean lengths of neurites showed no notable difference between control and OGD groups (Fig. 7A, B; 8A, B). Interestingly, when the co-culture was delayed to 48 h after OGD, reactive astrocytes inhibited neurite growth in both models. Neurons grew many long processes and most of them extended in the control groups (Fig. 7D, 8D), while many neurite processes were stunted, and some had abrupt turns or swollen endings in the OGD groups (Fig. 7D, 8D). Furthermore, control neurons had more connection and overlapping processes with surrounding neurons, but neurons in the OGD groups mostly appeared singly and lacked overlapping processes (Fig. 7A, 8A). The mean length of neurites in the OGD groups was reduced to 68.5±2.2% in the N model (Fig. 7B) and to 82.0±2.5% in the A+N model compared to control groups (Fig. 8B). To further study the inhibition of neurite growth was related to the expression of neurocan in astrocytes after OGD, chondroitinase ABC (chABC) was added into the co-culture system in N model, which digests glycosaminoglycan chains on CSPGs and can thereby overcome CSPGs, including neurocan, mediated inhibition. The neurite growth inhibition was significantly recovered by chABC, when the co-culture was performed at 48 h after OGD (Fig. 9B, D). Moreover, chABC even increased the neurite growth of neurons co-cultured at 24 h after OGD (Fig. 9A, C). However, chABC did not affect the neurite growth in both control groups (data not shown).

## Discussion

In the present study, we found that conditioned OGD (6 h) can induce astrocytes to present characteristics of glia scar after reperfusion, since astrocyte proliferation was increased and the expression of GFAP and neurocan were significantly upregulated following 24 h reperfusion. And these astrocytes play a dual role in neurite growth of neurons, as the neurons grew longer processes when the co-culture was performed immediately after OGD, but neurite growth was notably inhibited in two co-culture models when the co-culture was performed at 48 h of reperfusion after OGD. These phenomena mimic the effects of reactive astrocytes on nerve growth *in vivo*. Therefore, we considered that an *in vitro* model of glial scar induced by OGD was established for the first time, which is much useful for exploring the mechanisms underlying glial scar formation after stroke.

Cerebral ischemia leads to widespread progressive alterations in astrocytes, including cell hypertrophy, upregulation of intermediate filaments and increase in cell proliferation, which is commonly referred to as reactive astrogliosis [1]–[3], [15]. Here, we found that after 6 h OGD and an additional 24 h reperfusion, the expression of GFAP in astrocytes was significantly upregulated, and the number of BrdU immunopositive cells also notably increased in the OGD groups compared with controls. These results indicate that the astrocytes exhibit reactive astrogliosis in response to OGD during reperfusion. However, in our experiment, 6 h, but not 2 h, 4 h, 8 h or 12 h OGD caused mostly obvious proliferation of astrocytes, which suggests that the moderate injury is the necessary condition to induce glial scar-like changes of astrocytes. Such phenomenon is reasonable, because the reactive astrocytes and the glial scar formation exist in the penumbra, but not the core of the ischemia, where the blood flow is far lower than normal level [16]. In addition, following 24 h reperfusion after OGD, the expression of neurocan, a representative scar-associated hallmark [17]–[19], was remarkably increased. In our experiment *in vivo*, we also found that the expression of neuroncan showed an elevation at 7 d after cerebral ischemia, and the high expression spots of neurocan were well co-localized with astrocytes. Similar to our observations, other groups found that neurocan accumulates and upregulates around injuries responding to ischemia [19]–[22]. For example, Inatani et al. reported that neurocan transiently appears in the peri-ischemic region with a peak level at 4 days after tMCAO in rats [21]. Days to one week or even longer after ischemia is often considered as the subacute phase of the pathologic process after ischemia, during which the astrocytes are activated, proliferate and secrete many kinds of mediators including the neurocan [1]. Therefore, the 24 h reperfusion after OGD *in vitro* is possibly comparable to the subacute phase after ischemia *in vivo*, and study of the mechanism of astrocyte changes during this phase could also be carried out *in vitro*.

In addition, after stroke, the growth of neuronal axon is often arrested and the endings of axon are swollen [23]. Such bulbous axon terminals are considered signs of abortive regeneration, and have been labeled “dystrophic endings” [7]. In the present study, at 48 h reperfusion after OGD, the neurons exposed to OGD treated astrocytes showed abortive regeneration, as they often appeared singly with less overlapping, and had swollen terminals or abrupt turns in neurite processes, the growth of which was also inhibited. These morphologies are also consistent with neurons responding to an inhibitory environment induced by other injuries [7], [9], [11], [24]. Moreover, we found that the neurite growth inhibition was significantly reversed by chABC, which overcome the effect of CSPGs, including neurocan. Therefore, these data at least suggest that astrocytes at 48 h reperfusion after OGD in our experiment present a glial scar-like action to inhibit neuron growth. The astrocytes in other scar models induced by stretch [7] or mechanical scraping [11], also show scar-associated changes and present inhibitory effect on neuron growth, however which only occurs adjacently to the lesion border but not far away from the injuried tissues. Our present condition caused a more widespread area with scar-like patterns. Moreover, ischemia and traumatic insults arise from very different initial insults and lead to very different pathophysiology, which means mechanical injury models are not very suitable to study the pathologic mechanisms of ischemic insults. Thus, our ischemia-induced model *in vitro* will be helpful in exploring the mechanisms underlying glial scar formation caused by ischemia.

Interestingly, we found different results when the co-culture was performed at 0 h or 48 h of reperfusion. This indicated that reactive astrocytes play different roles in the acute phase and the later phase after OGD, which is similar to its functional roles *in vivo* [1], [15]. When the co-culture was made at the acute phase after OGD, the neurons on treated astrocytes had elongated neurites, which means OGD treated astrocyte improved neurite outgrowth. Growing evidences show that astrocytes exert a neuroprotective effect in the acute phase after stroke by shielding neurons from oxidative stress [5], providing trophic and metabolic supports to promote neuronal repair [25]–[27], or regulating excessive levels of glutamate, K^+^ and other ions [6]. These findings may help to explain how reactive astrocytes promote neuronal repair and survival in various ways during the acute phase after OGD, and also support that the changes of astrocytes in our system mimic those *in vivo*. On the other hand, when the co-culture was performed at 48 h of reperfusion, neurite growth inhibition occurred. But previous indexes to confirm the scar-like changes all were made at 24 h of reperfusion. This seems paradoxical, but is also interesting and complex. It may be attributed to the following two reasons. (i) Reactive astrogliosis is a finely graded continuum of progressive changes in gene expression and cellular changes [28]. It takes a time from synthesis to release of inhibitory proteins or cytokines by reactive astrocytes and from release to exerting an influence on neurons. And a delay of 24 h or more may be needed in our experiment. (ii) During the cerebral ischemia and reperfusion, astrocytes might either reduce or exacerbate the damage to neurons depending on the time point or post-ischemic stage [10], [29]. So, here may be due to the simultaneous presence of neurite growth-promoting and growth-inhibiting factors during 24 h reperfusion, which ultimately leads to the positive and negative effects balancing out. We also found that after treated with chABC, the neurite growth of neurons co-cultured at 24 h after OGD increased, which suggests that there exist grow-inhibiting factors, like CSPGs, in the co-culture system at 24 h after OGD.

The neurons in the in separated co-culture system showed more obvious change in neurite outgrowth than that in co-culture system with direct contact, as shown in Figs 7 and 8. In the acute phase after OGD, the mean length of neurites in the A+N model increased by 20% but more than 51% in the N model. During the recovery phase, the mean length of neurites in the N model was reduced to nearly 69% of controls, while it was reduced to 82% of controls in the A+N model. It is likely that the direct contact system is more stable and complex than the physically separated system and less vulnerable to many kinds of stimulation [30]–[31]. We suppose that the difference may be related to the existence of some factors, which are released or buffered by astrocytes only in contact co-culture system, playing different roles in acute and recovery phase after OGD, and further study is needed to elucidate the detailed mechanism.

Phosphacan, another major component of the CSPG family [15], [32], [33], is strongly up-regulated following various kinds of CNS injury *in vivo*, such as spinal cord injury [9], trauma [8] and ischemia [34], [35]. It also inhibits neurite outgrowth from different populations of neurons [36]–[39]. So, many studies regard phosphacan as a hallmark of glial scar [7], [32]. However, to our surprise, we found that phosphacan was reduced at 24 h after OGD and recovered to the control level at 72 h. Similar to our results, after hypoxia-ischemia, the protein level of phosphacan is reduced [19], [40]. Jones et al. also reported that it decreases immediately but later recovers and then peaks after 2 months following spinal cord injury [33]. Considering the varying expression according to different intensities of cellular response and various kinds of CNS injury, it seems that phosphacan is not an appropriate marker of glial scar.

In conclusion, we found that after conditioned OGD, the astrocytes presented the characteristics of the glial scar, which are also comparable to the astrocytes in acute and chronic phases after cerebral ischemia *in vivo*. Therefore, the present system may be used as an *in vitro* model to explore the mechanisms underlying glial scar formation and the treatments to improve axonal regeneration after cerebral ischemia, but direct evaluation for drugs has not been performed. Moreover, the glial scar-like change of astrocytes may not be persistent as that *in vivo*, and the time window to induce glial scar-like change seems narrow, so strictly controlling the experimental condition and examining the glial scar formation index at the same time are necessary during each test.

## Methods

### Ethics and Statement

All procedures were approved by the Zhejiang University Animal Experimentation Committee (Zju2009-1-02-004) and conformed to the National Institutes of Health Guide for the Care and Use of Laboratory Animals. All efforts were made to minimize the number of animals used, and their suffering.

### Primary Cortical Astrocyte Cultures

Primary cortical astrocyte cultures were prepared from 24 h postnatal Sprague-Dawley rats as described previously [41]. Briefly, the cerebral cortices were digested with 0.25% trypsin for 15 min at 37°C, and then the dissociated cells were seeded onto poly-D-lysine-coated 25 cm^2^ flasks. Cells were cultured in Dulbecco’s Modified Eagle Medium (DMEM, Invitrogen, USA) supplemented with 10% fetal bovine serum, 2 mM L-glutamine, 100 units/ml penicillin and 100 µg/ml streptomycin. The cultures were maintained at 37°C under >90% humidity and 5% CO_2_. The medium was changed every 2–3 days until cells reached confluence. After 12–14 days, the confluent cultures were shaken overnight to minimize microglia contamination. The remaining astrocyte monolayers were trypsinized and replated at a density of 2.5×10^4^ cells/cm^2^. More than 95% of the cultured cells were astrocytes as identified by immunofluorescent staining for GFAP.

### Oxygen-glucose Deprivation

Oxygen-glucose deprivation was performed as previously described [19]. Briefly, astrocytes were incubated in DMEM containing 2 mM L-glutamine, 100 units/ml penicillin, 100 µg/ml streptomycin and 1% fetal bovine serum for 16 h, and then washed twice and incubated in glucose-free DMEM (Invitrogen, USA). Then the cultures were transferred into an anaerobic chamber filled with a gas mixture of 95% N_2_/5% CO_2_ at 37°C. At the end of the OGD, astrocytes were incubated in DMEM containing 2 mM L-glutamine, 100 units/ml penicillin, 100 µg/ml streptomycin and 1% fetal bovine serum, and reintroduced to the regular atmospheric oxygen level for an additional 24, 48 or 72 h. In each experiment, cultures exposed to OGD were compared with normoxic controls supplied with DMEM containing glucose and maintained in standard incubation conditions.

### Primary Cortical Neuron Cultures

Primary cortical neurons were prepared from embryonic day 18 Sprague-Dawley rats as described previously [42] with the following modifications. In brief, cortices were dissected from the brains under sterile conditions, digested in 0.25% trypsin in Hank’s balanced salt solution for 15 min at 37°C and then mechanically dissociated. The cells were spun down for 5 min at 1000 g and resuspended in Neurobasal medium (Invitrogen, USA). Cells were seeded at a density of 1.5×10^5^ cells/cm^2^ to glass coverslips coated with poly-D-lysine (0.1 mg/ml, Sigma, USA). Neurobasal medium was supplemented with 100 units/ml penicillin, 100 µg/ml streptomycin, 0.5 mM L-glutamine and 2% B-27. The cultures were maintained in a humidified CO_2_ incubator (5% CO_2_, 95% air, 37°C). Neurons were used for immunocytochemistry after co-culture with astrocytes for 24 h.

### Astrocyte and Neuron Co-culture

Two co-culture methods were used in this system. In method A (termed N), astrocytes were subcultured on Petri dishes and subjected to OGD for 6 h. After different reperfusion times (0 h, 24 h, 48 h), four glass coverslips were placed on the bottom of the dish. Then neurons were seeded on the coverslips, and cultured for additional 24 h. To study the role of neurocan in the inhibition of neurite growth, chABC at 0.2 U/ml was added at 2 h before the seeding of neurons. In this method, neurons and astrocytes are physically separated, but the medium was intermixed. In method B (termed A+N), at first, glass coverslips were placed on the bottom of a Petri dish. Astrocytes were subcultured on coverslips and subjected to OGD for 6 h. After different reperfusion times (0 h, 24 h, 48 h), neurons were seeded on the coverslips, and cultured for additional 24 h. In this method, neurons and astrocytes have direct contact. All the Petri dishes and glass coverslips were coated previously with poly-D-lysine (0.1 mg/ml). The controls were co-cultured with astrocytes, which had not been exposed to OGD.

### MTT Assay and Cell Apoptosis and Necrosis Study

For MTT assay, astrocytes were subcultured on 96-well plates. The cells were incubated with MTT (Sangon, China) 0.5 mg/ml as final concentration for 2 h at 37°C. Then, the supernatant layer was removed, and 100 µl of dimethyl sulfoxide was added to each well. MTT metabolism was quantified spectrophotometrically at 570 nm in a Biorad microplate reader. Results were expressed as the percentage of MTT reduction, assuming the absorbance of control cells was 100%. Moreover, Hoechst 33342 and propidium iodide double staining was performed to evaluate the cell apoptosis and necrosis after OGD [43]. A cell that shows nuclear condensation and DNA fragmentation detected by Hoechst 33342 is undergoing apoptosis, while a cell that is permeable to propidium iodide is undergoing necrosis.

### Immunocytochemistry

Immunostaining was also performed in cultured astrocytes and neurons as previously described [44]. Cells seeded on coverslips were fixed with 4% paraformaldehyde for 15 min and incubated in 3% bovine serum albumin (BSA) containing 0.1% Triton X-100 at 37°C for 1 h. Then primary antibodies in 3% BSA were applied for 0.5 h at 37°C. For astrocytes, rabbit anti-GFAP (1∶100, Zhongshan, China), rabbit anti-neurocan (1∶200, Boster, China) and mouse anti-phosphacan (1∶100, DSHB, USA) were used. For neurons, mouse anti-class III β-tubulin (1∶2000, Sigma, USA) was used. After repeated washes in phosphate-buffered saline (PBS), cells were incubated with secondary antibody in 3% BSA for 0.5 h at 37°C. The secondary antibodies used in this experiments included goat anti-rabbit IgG-Alexa 488 (1∶300, Invitrogen, USA), goat anti-mouse IgG-Alexa 488 (1∶300, Invitrogen, USA) and goat anti-mouse IgG-Alexa 594 (1∶300, Invitrogen, USA). For actin staining, cells were incubated in rhodamine-phalloidin (Molecular Probes; 1∶50, USA) at the same time. After further washing in PBS, cultures were dried, coverslipped, and mounted onto glass slides. Finally, the stained cells were observed under fluorescent microscopy (Olympus BX51, Japan). The immunofluorescence intensity of GFAP, neurocan and phosphacan for each field was analyzed by NIH Image J software and then normalized by the cell number. Five to eight random fields were analyzed for each coverslip in every independent experiment, which was replicated for 3–6 times.

### Neurite Growth Quantification

The lengths of neurites in each neuron were traced manually and quantified by NIH Image J software. Only cells with a valid nucleus and cell body (class III β-tubulin-positive) were included in the analysis. Mean neurite length for each neuron was determined for 50–200 neurons per condition from three to six separate experiments.

### Proliferation Assay

To assess proliferation of glial cells in culture, cells were incubated with 10 µM BrdU during OGD, for 24 h after OGD, or for 24 h after 24 h reperfusion of OGD, after which the cells were fixed with 100% ice-cold methanol for 10 min at 4°C. And then the cells were washed in PBS for 3 min, and the DNA was denatured by incubating in 2 M HCl at 37°C for 1 h. After washed twice in 0.1 M borate buffer (pH 8.5) and three times in PBS, cells were blocked with 3% BSA at 37°C for 1 h. After that, cells were incubated in mouse monoclonal antibody against 5-bromodeoxyuridine (BrdU, 1∶2000, Sigma, USA) at 37°C for 0.5 h and then washed twice with PBS. Cells were then incubated with goat anti-mouse IgG-Alexa 594 (1∶300, Invitrogen, USA) at 37°C for 0.5 h. After further washing in PBS, cells were mounted, and observed under fluorescent microscopy (Olympus BX51, Japan). Cell number was counted by NIH Image J software.

### Cerebral Ischemia and Immunohistochemistry

Adult male Spraque Dawley rats (250–280 g) were anesthetized with chloral hydrate (350 mg/kg), and subjected to transient middle cerebral artery occlusion (tMCAO). In brief, a silicone-coated suture with round tip was inserted into the right internal carotid to enter the origin of the middle cerebral artery. After occlusion for 90 min, the suture was withdrawn to induce reperfusion. After different during of reperfusion, the penumbra region of the rats were separated for Western blot analysis, or the rats were transcardially perfused by 0.9% cold saline and 4% paraformaldehyde. The brains were separated and stored in 4% paraformaldehyde at 4°C for 24 h, and then in 30% sucrose for 3 d. The dissected brains were then cut into 10 µM coronal cryosections by a cryostat (SM2000R, LEICA, Germany) and immunostained for neurocan and GFAP. For fluorescent immunostaining, the sections were first incubated with 3% BSA in PBS for 30 min, and then with rabbit anti-neurocan (1∶100, Boster, China) and mouse anti-GFAP (1∶300, Millipore, USA) in PBS containing 0.3% Triton X-100 at 4°C overnight. After washing three times for 10 min with PBS, sections were incubated sequentially in goat anti-rabbit IgG-Alexa 488 (1∶200) and goat anti-mouse IgG-Alexa 594 (1∶300, Invitrogen, USA) serum for 2 h at room temperature. For diaminobenzidine (DAB) histochemistry staining, endogenous peroxidases were quenched by treatment with 3% H_2_O_2_ in methanol, and slides were blocked with 10% normal goat serum. Slides were then stained with rabbit anti-neurocan (1∶200, Boster, China) and were processed by using Histostain-Plus IHC Kit (MR Biotech, China) and hemotoxylin counterstain (KeyCEN BioTECH). Then the sections were mounted after washing three times for 10 min with PBS. Images of penumbra region in the stratium were captured under fluorescent microscopy (Olympus BX61, Japan).

### Western Blot

For neurocan and phosphacan expression after OGD, identical amounts (500 µL) of astrocyte supernatant, after various treatments, were concentrated by Amicon Ultra-0.5 ml Centrifugal Filters (100 kD, Millipore) and centrifugation at 10000 g, 4°C for 25 min. For the neurocan expression after tMCAO in rats, total proteins in the penumbra were purified using cell and tissue protein extraction reagents (Kangchen, Shanghai, China). Protein concentration was determined with the bicinchoninic acid assay (Pierce, USA). Then protein samples were digested with 0.5 U/ml chondroitinase ABC (Chase ABC, Sigma, USA) in Tris buffer (50 mM Tris, pH 8.0, 60 mM sodium acetate and 0.02% bovine serum albumin) at 37°C for 1.5 h, and then denatured with loading buffer for 5 min. Protein samples were separated on 7.5% SDS-polyacrylamide gels and then electrotransferred onto a nitrocellulose membrane. After blocking with 5% fat free milk, the membranes were incubated with rabbit anti-neurocan (1∶400, Boster, China), mouse anti-phosphacan (1∶1000, Sigma, USA), rabbit anti-GFAP (1∶300, Boster, China) or mouse anti-glyceraldehyde-3-phosphate dehydrogenase (anti-GAPDH, 1∶5000, Kangchen, Shanghai, China) at 4°C overnight. After repeated washes, the membranes were reacted with IRDye 800 anti-rabbit Molecular Probe (1∶8000, LI-COR Biosciences, USA) or IRDye 700 anti-mouse Molecular Probe (1∶3000, LI-COR Biosciences, USA) for 2 h. Images were acquired with the Odyssey infrared imaging system (LI-COR Biosciences, USA) and analyzed by the software as specified in the Odyssey software manual.

### Statistical Analysis

All data represent three or more independent experiments. Data are presented as mean ± SEM. Two tailed-Student’s *t*-test was applied for comparisons between control and OGD group. One-way ANOVA followed by the LSD or Dunnett’s T3 post-hoc test (where equal variances were not assumed) was applied for multiple comparisons. *P*<0.05 was considered statistically significant.
